# A pre-operative elevated neutrophil: lymphocyte ratio does not predict survival from oesophageal cancer resection

**DOI:** 10.1186/1477-7819-8-1

**Published:** 2010-01-06

**Authors:** Farhan Rashid, Naseem Waraich, Imran Bhatti, Shopan Saha, Raheela N Khan, Javed Ahmed, Paul C Leeder, Mike Larvin, Syed Y Iftikhar

**Affiliations:** 1 Royal Derby Hospital, Uttoxeter Road, Derby, DE22 3NE, UK; 2 School of Graduate Entry Medicine and Health, Derby, University of Nottingham, Uttoxeter Road, Derby, DE22 3DT, UK; 3 Academic Division of Upper GI Surgery, School of Graduate Entry Medicine and Health, University of Nottingham, The Medical School Derby, DE22 3DT, UK

## Abstract

**Background:**

Elevated pre-operative neutrophil: lymphocyte ratio (NLR) has been identified as a predictor of survival in patients with hepatocellular and colorectal cancer. The aim of this study was to examine the prognostic value of an elevated preoperative NLR following resection for oesophageal cancer.

**Methods:**

Patients who underwent resection for oesophageal carcinoma from June 1997 to September 2007 were identified from a local cancer database. Data on demographics, conventional prognostic markers, laboratory analyses including blood count results, and histopathology were collected and analysed.

**Results:**

A total of 294 patients were identified with a median age at diagnosis of 65.2 (IQR 59-72) years. The median pre-operative time of blood sample collection was three days (IQR 1-8). The median neutrophil count was 64.2 × 10^-9^/litre, median lymphocyte count 23.9 × 10^-9^/litre, whilst the NLR was 2.69 (IQR 1.95-4.02). NLR did not prove to be a significant predictor of number of involved lymph nodes (Cox regression, p = 0.754), disease recurrence (p = 0.288) or death (Cox regression, p = 0.374). Furthermore, survival time was not significantly different between patients with high (≥ 3.5) or low (< 3.5) NLR (p = 0.49).

**Conclusion:**

Preoperative NLR does not appear to offer useful predictive ability for outcome, disease-free and overall survival following oesophageal cancer resection.

## Introduction

Human oesophageal carcinoma is considered one of the most aggressive malignancies and is associated with a poor prognosis [1]. Despite recent advancement in surgical and oncological treatment the five year survival remains very poor [2-4]. Oesophagectomy for oesophageal cancer is a major operative intervention which carries a high risk of complications. Hence any means of predicting patients with an inherently poor prognosis or high risk from surgery would be valuable in making treatment recommendations.

Generally agreed prognostic factors for most gastrointestinal cancers include tumour size, marginal resection line involvement, lymph node metastases and tumour differentiation [5]. During the last fifteen years there has been debate about the interaction between cancer and host inflammatory responses, in particular whether cancer may alter regulation leading to further DNA damage, promotion of angiogenesis, inhibition of apoptosis and increased metastastic susceptibility [6-10]. It is clear that the response of the immune system plays a vital role in the control and progression of many disease states including cancer. Simple measures of immune responsiveness include simple routine biochemical and haematological markers such as total and differential leukocyte counts and C-reactive protein (CRP), which have been proposed as diagnostic and prognostic factors for a variety of cancers [11,12]. This may permit a simple estimate of inflammatory response to cancer which is easily assessed in everyday clinical practice.

CRP is the most commonly used measure of systemic inflammation in clinical practice, and has been shown to be an independent predictor of survival in patients undergoing resectional surgery for colorectal cancer [13,14]. Haematological factors which have been scrutinised for prognostic value include lymphocyte count, neutrophil count and neutrophil: lymphocyte ratio in patients undergoing surgery for pancreatic ductal cancer, epithelial ovarian cancer and hepatic resection of colorectal liver metastases [15,11,16]. The effect does not appear to be restricted to major surgical interventions as an elevated NLR has also been shown to predict a poor outcome from interventional procedures for vascular and cardiovascular diseases [17,18].

All patients undergoing oesophagectomy have preoperative full blood counts taken routinely. The NLR can be calculated easily from the data already available. NLR and other inflammatory markers have been identified as a predictor of outcome in patients undergoing potentially curative resection for other gastrointestinal cancers, including hepatocellular and colorectal carcinoma [13,15,16,19]. The role of NLR in patients undergoing oesophageal cancer resection does not yet appear to have been studied. The present study was carried out to examine the hypothesis that an elevated pre-operative NLR might prove a clinically useful prognostic indicator for post-operative survival and disease free interval following oesophageal cancer resection. Prognosis would be assessed against standard clinical and histopathological data.

## Materials and methods

### Study subjects

A retrospective analysis was carried out in accordance with UK clinical research governance guidelines, and was approved by our institutional audit department. Patients who underwent surgical resection for oesophageal cancer from June 1997 to September 2007 were identified from our local database for oesophageal cancer. Demographic details, pre-operative staging data, operation type, histopathological diagnosis, staging and survival were extracted from the database. Pathological staging was determined using the American Joint Committee on Oesophageal Cancer staging, which stages tumours according to a revised tumour node metastasis (TNM) system. All patients were followed up in out-patient clinics at regular intervals. First follow up was undertaken at 6 weeks following surgery and subsequently after 3 months, 6 months, 9 months, 1 year and thereafter at every six months interval. Survival data was analysed in October 2007.

### Calculation of Neutrophil lymphocyte ratio

Routine full blood count (FBC) results were collected as part of standard diagnostic and pre-operative protocols. The NLR was calculated as a simple ratio between the absolute neutrophil and the absolute lymphocyte counts, as provided from the differential white cell count output from a standard Coulter^® ^counter (Model, XE2100, Sysmex, Japan).

### Statistical methods

The distribution of continuous variables was tested for normality using the Kolmogorov-Smirnov test and Q-Q plots. All continuous variables were skewed therefore the results were reported as medians {Interquartile range (IQR)}. The Spearman's correlation coefficient was used to assess the association between continuous variables. The Mann-Whitney U test was calculated for comparison of two groups and the Kruskal-Wallis test was used to compare more than two groups. Cox regression and Kaplan-Meier analysis was utilised to assess the predictive value for NLR, neutrophil and lymphocyte counts for hazard of death. The Kaplan-Meier curves were compared using the Log Rank test. The Cox regression models were constructed using the Forward: Likelihood ratio method with p value less than 0.05 as the entry criterion to the model for the independent variables. The hazard risk (HR) from the Cox Regression analysis was not presented for non significant specific variables that were tested. The Chi-Square test was used to test the association between NLR groups (Cut-offs of 3, 3.5, 4 and 5) and recurrence, Tumour (T)-stage, Nodal (N) stage and histological subtype of cancer.

SPSS version 16.0 was used for statistical analysis (SPSS, Woking, Surrey UK). An alpha probability (p value) of less than 5% (0.05) was considered significant.

## Results (Table 1)

**Table 1:** Demographics and preoperative haematology results from patients with resected oesophageal cancer.

*Demographics*	
	

No of patients identified	294

Male/Female	235:59

Median age (IQR)	65.2 (59-72) years

	

Overall median survival (IQR)	22 (14-90) months

	

Histological subtypes	

Adenocarcinoma	238(81%)

Scquamous cell carcinoma	50(17%)

	

	

	

Preoperative FBC available	294

Median neutrophil count (IQR)	64.2 × 10^-9^/litre, (58-71)

Median lymphocyte count (IQR)	23.9 × 10^-9 ^/litre, (17-30)

Neutrophil lymphocyte ratio(IQR)	2.69,(1.95-4.02).

Median timing of preoperative FBC (IQR)	3 (1-8)

	

Neutrophilia (> 7.5×10^6^/ml)	265(94%)

Lymphocytopenia (< 1.0 ×10^6^/ml)	57(20%)

Of 294 patients studied, there were 235 males and 59 females. The median age at diagnosis was 65.2 years (IQR 59-72). There were 238 adenocarcinomas (81%), 50 squamous cell cancers (17%) and 6 other cancers (2%) comprising 2 gastrointestinal stromal tumours, one oat cell cancer and three undifferentiated oesophageal tumours. The median time for pre-operative FBC sample collection was 3 days, (IQR: 1 - 8). No patient exhibited clinical signs of sepsis in the pre-operative period.

### Neutrophil: lymphocyte ratio (Table 1)

The overall median neutrophil count was 64.2 × 10^-9^/litre, IQR 58.6-71.0, the median lymphocyte count 23.9 × 10^-9 ^/litre, IQR 17.8-30.0 and the NLR was 2.69, IQR 1.95-4.02).

### NLR as a predictor of death

NLR was not a significant predictor of hazard of death (Cox Regression analysis, p = 0.374).

### NLR and age

There was no significant correlation between age and NLR (r = 0.094, p = 0.117, Spearman's correlation coefficient).

### Neutrophil: lymphocyte ratio in cancer subsets (Figure 1) (Table 1)

**Figure 1:**
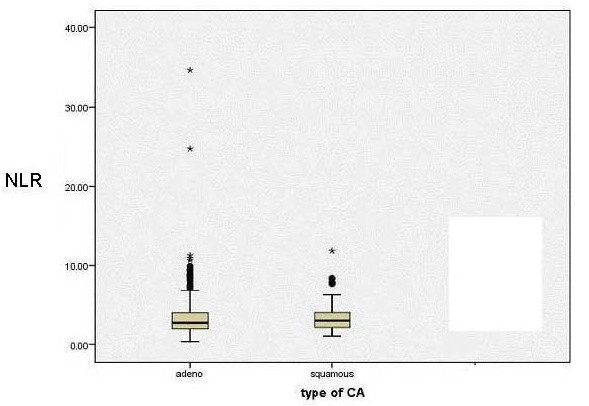
**NLR median and IQR box plot for three oesophageal cancer groups**.

NLR values were not significantly different between patients within the two different types of cancer (adenocarcinoma 2.69, IQR 1.32-3.96 and squamous cell carcinoma 2.98, IQR 2.10-4.10. Mann Whitney U test p = 0.740) (Figure 1).

### NLR and nodal status

**Figure 2:**
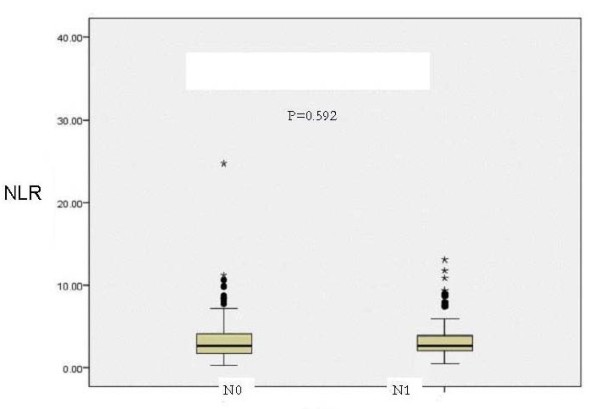
**NLR value and TNM nodal status**.

**Figure 3:**
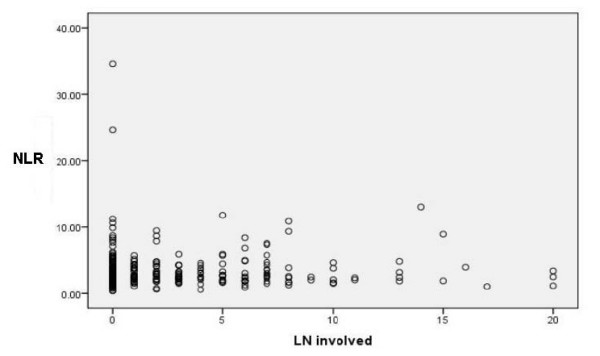
**NLR and ratio of involved to total lymph node yields**.

NLR values were not significantly different between TNM subsets of lymph node status. The median NLR in pN0 (no lymph node metastasis) patients was 2.69, IQR 1.75-4.10 and in pN1 (regional lymph node metastasis) patients was 2.69, IQR 2.08-3.93, which was not significantly different (p = 0.592). (Figure 2). NLR value was not significantly correlated with either the lymph node yield, (r = 0.28, p = 0.644) nor with the involved lymph node (r = 0.42, p = 0.493) (Figure 3).

### NLR and T stage

There was no relationship between different NLR cut off values (3, 3.5,4 and 5) and the depth of invasion or T stage (p values of 0.624, 0.937, 0.866 and 0.522 respectively).

### NLR and disease recurrence (Table 2)

**Table 2:** NLR values and disease recurrence

	NLR	IQR	P-Value
Recurrence Free	2.82	1.78-4.07	0.288

Recurrence	2.79	212-4.28	0.288

There was no significant relationship between NLR values and the probability of disease recurrence (recurrence free 2.82 IQR1.78-4.07, P = 0.82, and recurrent disease 2.79 IQR 2.12-4.28, P = 0.288).

### NLR vs Survival (Figure 4, 5, 6 &7)

**Figure 4:**
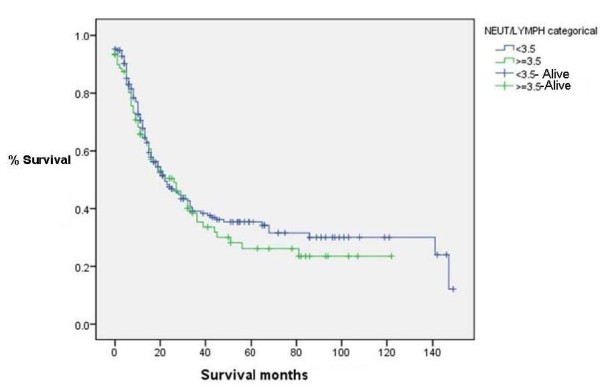
**Survival for patients with NLR < 3.5 and > = 3.5(Censored = alive) (p = 0.49)**.

**Figure 5:**
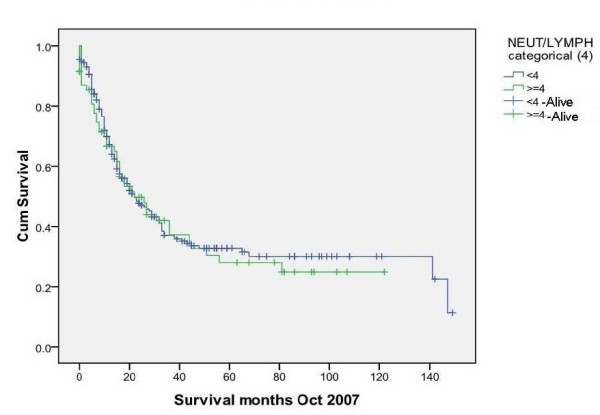
**Survival for patients with NLR < 4 and > = 4(Censored = alive) (p = 0.680)**.

**Figure 6:**
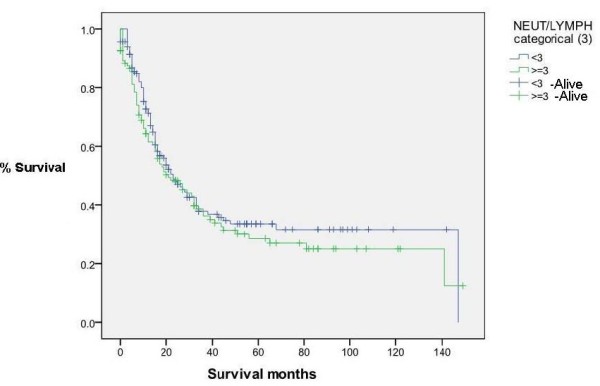
**Survival for patients with NLR < 3 and > = 3(Censored = alive) (p = 0.340)**.

**Figure 7:**
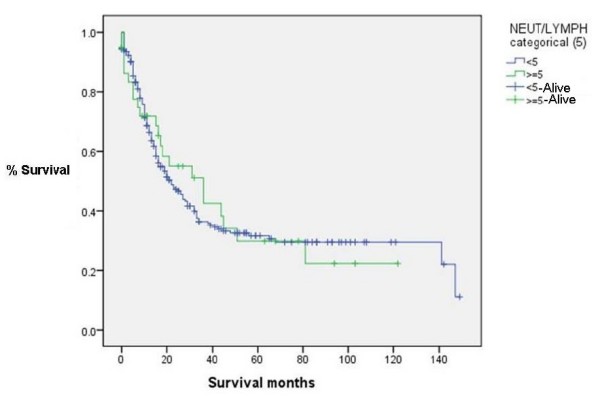
**Survival for patients with NLR < 5 and > = 5(Censored = alive) (p = 0.868)**.

NLR was grouped into different cut-off points (3, 3.5, 4 and 5) to find whether there was any significant difference in survival. There was no significant difference in survival between patients with NLR values of greater than or equal to 3.5 and those with an NLR of less than 3.5. The median overall survival was 22 months, IQR 14-90. Survival time was not statistically significantly different between groups with NLR ≥ 3.5 and those with < 3.5 (*p *= 0.49). Similarly, the choice of other NLR cutt offs (3, 4 and 5) did not show any significant difference in survival (p values of 0.340, 0.680 and 0.868 respectively).

### NLR and preoperative chemotherapy

Fourty four patients had preoperative chemotherapy as compared to 250 patients who underwent surgery as first line treatment without neo-adjuvant chemotherapy. The neutrophil count for patients with chemotherapy (Median 57.8, (49-64.7)) was lower than patients without chemotherapy (Median 65.3, (60-72), p < 0.001). However, the patients with chemotherapy had higher lymphocyte count (Median 31, 22-37) as compared to those without preoperative chemotherapy (Median 22.7 (17-28.6), p < 0.001). Median NLR of those who had chemotherapy was 1.86 (IQR, 1.3-2.9) and those without chemotherapy was 2.8 (IQR, 2.1-4.3) (p < 0.001). There was no survival difference in patients with or without chemotherapy (p = 0.323). In addition, adjusting for NLR, there was no difference in survival for patients who had received preoperative chemotherapy as compared to those without neoadjuvant chemotherapy (p = 0.280, Cox regression analysis with interaction term).

### NLR cut off values and type of cancer (Table 3)

**Table 3:** Association between the type of cancer and NLR > = 3.5 and < 3.5

p = 0.984 (Chi Square test)		NLR < 3.5	> = 3.5	Total
**Adeno**	**n**	165	71	236
	%	81.3%	80.7%	81.1%

**Squamous**	**N**	34	15	49
	%	16.7%	17.0%	16.8%

Different values of NLR have been used as a predictor of prognosis [15]. A cut off value 3.5 has also not shown any significant association between two sub-types of oesophageal cancer and NLR values.

## Discussion

Leukocytes were first discovered in malignant tissue specimens by the pathologist Rudolf Virchow about 150 years ago [6]. Inflammation not only plays a vital role in development but also remains very important in progression of various malignant disease processes including gastrointestinal tract [20-22] and liver cancers [23]. Neutrophilia has been associated with malignancy, although the cause is not completely understood. However, it is a multifactorial process. Research has confirmed a link between the inflammatory microenvironment of a tumour, and systemic responses induced by the tumour. The presence of T-cells in a tumour provides an indication of significant local immune responses [24,25]. For many types of cancer, lymphocytopaenia indicates a generalized state of immunodepression [26], and survival appears to be adversely influenced by depressed immune function. There may be a marked decrease in CD-4 helper lymphocytes and an increase in CD-8 suppressor lymphocytes, signifying depression of innate cellular immunity [27]. Depression in T-cell function may attenuate the tumour specific response. Major surgery in cancer patients is known to reduce lymphocyte metabolism, as measured by adenosine triphosphate production, which leads to functional impairment [28]. In addition, the microenvironment within the tumour can also influence on the invading leukocytes to enhance angiogenesis, invasion, motility and viability [6,7,29,30].

The malignant process also produces myeloid growth factors as part of a paraneoplastic syndrome and this may be one of the causes of neutrophilia. In addition, another factor granulocyte colony stimulating factor produced by the malignant cells has also been attributed to be the cause of neutrophilia because of its action on bone marrow granulocytic cells [31-35]. Apart from the production of myeloid growth factors, cancer inflammation and associated neutrophilia have also been associated with the release of IL-6 (interleukin-6) and TNF-α (Tumour necrosis factor-α) [36-39].

Some variations have been observed in different cancers. Patients with pancreatic ductal adenocarcinoma have been identified as having more marked lymphocytopenia preoperatively and postoperatively, when compared with patients having gastric and colorectal carcinoma [39]. Previous studies have suggested different NLR values as a prognostic marker [15,17,40]. The preoperative NLR of greater than 5 was elevated in only around 15% of our patients as compared to 32% in the study published by Walsh et al in patients with colorectal cancer. In addition, majority of the patients (94%) in our study had neutrophilia as compared to near normal neutrophil count in most of the patients undergoing resection of pancreatic ductal adenocarcinoma [16].

The oesophageal tumour occurs more frequently in males and such tumours have a worst prognosis when compared to their female counterparts [1]. The gender effects on the changes of circulating subtypes of white cells, the differences in endocrine reactions to the nature of the stress have been studied and certain variations in immune response between males and females have also been reported [41-44]. The females have shown a more immunocompromised response as compared the male patients [41]. Although the immune response is multifactorial, the male predominance of oesophageal cancer (male to female ratio of 4:1 in this study) may be one of the reasons why NLR does not work as a predictor in our study as compared to the other studies.

All these factors may explain the variance in the results of our study compared to others undertaken in different cancers.

**Figure 8:**
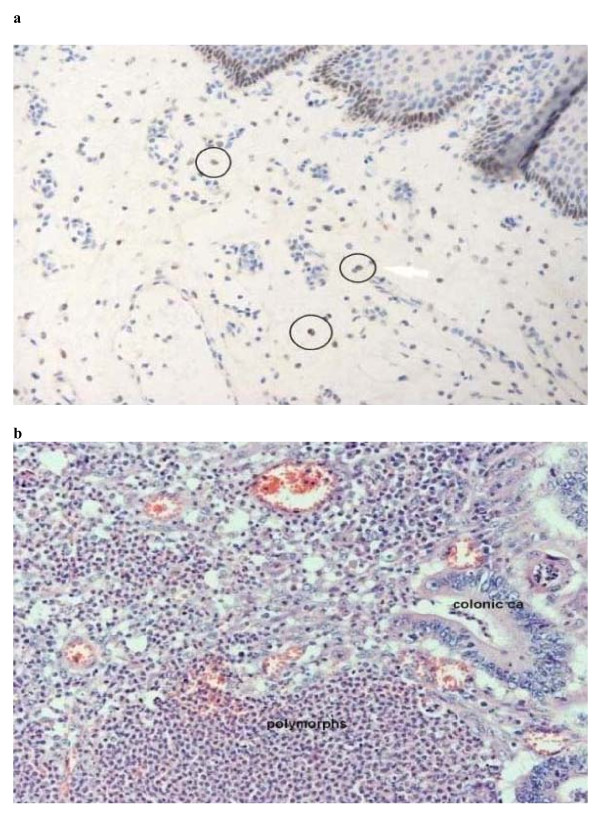
**a Oesophageal cancer histopathology: there is marked dysplastic change but little polymorphic infiltration**. **8b: **Colonic tumour with excessive polymorphic infiltration but little dysplasia.

Inflammation is known to play a role in some colorectal cancers. This includes causation, with ulcerative colitis known to involve recurrent ulceration, epithelial regeneration dysplasia and in some cases malignant change. Oesophageal cancer can be preceded by Barrett's oesophagus, also a chronic inflammatory process involving metaplasia (figure 8a &8b). However the majority of gastrointestinal tract cancers do not arise as a result of overt acute or chronic inflammation. Nevertheless, cancer invokes a host inflammatory reaction as a consequence.

Immunosurveillance for cancer fails as humans age [45,46], and this may also explain changes in neutrophil and lymphocyte counts in oesophageal cancer, predominantly a disease of older patients. 58% of our patients were over 60 years of age, in keeping with most published series.

Our cohort includes only those oesophageal cancer patients who had resectable disease and underwent surgery and does not include those who underwent palliative treatment. This exclusion of the patients with metastatic disease remains a shortcoming of the study.

In conclusion, the present study failed to confirm that NLR was a significant predictor of survival, recurrence and nodal involvement following resection for oesophageal cancer.

## Abbreviations

CRP: C-reactive protein; CA: carcinoma; DNA: deoxyribonucleic acid; FBC: full blood count; IQR: interquartile range; LN: lymph node; NLR: neutrophil lymphocyte ratio.

## Conflict of interests

The authors declare that they have no competing interests.

## Authors' contributions

FR has designed, carried out the study. FR and NW helped in data collection. FR, NW and IB have performed the analysis. JA, PCL, MLA and SYI provided the supervision. FR wrote the manuscript. PCL, RNK, MLA and SYI edited the manuscript. All authors contributed to the manuscript, and all read and approved the final version.
